# Fatigue in Aviation: Safety Risks, Preventive Strategies and Pharmacological Interventions

**DOI:** 10.3389/fphys.2021.712628

**Published:** 2021-09-06

**Authors:** Yara Q. Wingelaar-Jagt, Thijs T. Wingelaar, Wim J. Riedel, Johannes G. Ramaekers

**Affiliations:** ^1^Center for Man in Aviation, Royal Netherlands Air Force, Soesterberg, Netherlands; ^2^Department of of Neuropsychology and Psychopharmacology, Faculty of Psychology and Neuroscience, Maastricht University, Maastricht, Netherlands; ^3^Diving Medical Center, Royal Netherlands Navy, Den Helder, Netherlands

**Keywords:** sleep, wakefulness-promoting agents, modafinil, caffeine, hypnotics and sedatives, temazepam, aerospace medicine, pilots

## Abstract

Fatigue poses an important safety risk to civil and military aviation. In addition to decreasing performance in-flight (chronic) fatigue has negative long-term health effects. Possible causes of fatigue include sleep loss, extended time awake, circadian phase irregularities and work load. Despite regulations limiting flight time and enabling optimal rostering, fatigue cannot be prevented completely. Especially in military operations, where limits may be extended due to operational necessities, it is impossible to rely solely on regulations to prevent fatigue. Fatigue management, consisting of preventive strategies and operational countermeasures, such as pre-flight naps and pharmaceuticals that either promote adequate sleep (hypnotics or chronobiotics) or enhance performance (stimulants), may be required to mitigate fatigue in challenging (military) aviation operations. This review describes the pathophysiology, epidemiology and effects of fatigue and its impact on aviation, as well as several aspects of fatigue management and recommendations for future research in this field.

## Introduction

“My mind clicks on and off … I try letting one eyelid close at a time while I prop the other open with my will. My whole body argues dully that nothing, nothing life can attain, is quite so desirable as sleep. My mind is losing resolution and control.” (Lindbergh, 1953).

That description in 1953 by Charles Lindbergh of his historic solo transoceanic flight of 33.5h in 1927 illustrates the destructive effects of fatigue in aviation. He was not the first to identify fatigue as a risk factor for aviation accidents. For example, in 1938, the Civil Aeronautics Act addressed the issue of aircrew duty hours and flight times (United States Government, 1938) However, throughout the years, fatigue remained an important risk factor for aircraft incidents and accidents both in civil and military aviation. In the last two decades, it has been identified as the probable cause of 21–23% of major aviation accidents investigations (Caldwell, 2012; Marcus and Rosekind, 2017; Gaines et al., 2020). In 2020, the European Aviation Safety Agency (EASA) identified “state of wellbeing and fitness for duties” as top safety issue for large aeroplanes (European Union Aviation Safety Agency (EASA), 2020).

The optimal method of avoiding fatigue is to have sufficient (night-time) sleep. This is often difficult to achieve in aviation, however, especially during military deployments, as sleep in the field is often of lesser quality and duration than sleep at home (Kelley et al., 2018). Moreover, performing operations at night may be tactically necessary. This can lead to irregular sleep during deployment, which may cause fatigue. This may be problematic particularly at the end of flight missions, as the landing phase has been identified as a risk factor for the occurrence of aviation accidents (European Union Aviation Safety Agency (EASA), 2020). The variety of aircrafts and types of operations performed by the Royal Netherlands Air Force (RNLAF), and the subsequent diversity, for example in duty periods and crew composition, have made it challenging to introduce appropriate Flight Time Limitations (FTL). This, together with the possibility of deviating from these regulations in case of operational necessity, has contributed to the impossibility of relying solely on these limitations to manage fatigue. Other countermeasures are therefore needed to enhance the fitness of pilots to fly under these circumstances. Currently, the RNLAF allows its pilots to use certain hypnotics to get sufficient sleep (Military Aviation Authority, 2021). Another solution is to prescribe stimulants, i.e., medications that increase vigilance and diminish fatigue. Although caffeine is widely available, both in pills and beverages, many aircrew members have reported that caffeine supplements are ineffective, which might be due to high daily caffeine consumption (Chou et al., 1985). Other pharmaceutical agents, e.g. (dextro) amphetamines and modafinil, are available, but their effects on (military) aviation are not always clear. Before implementing new countermeasures, the RNLAF requested a review of the current available literature.

**Figure 1:**
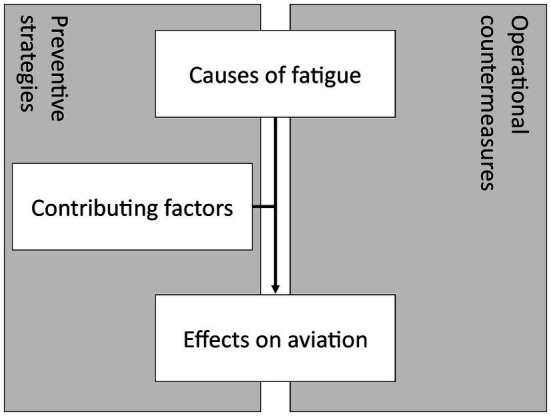
Root cause analysis of fatigue and areas of influence of fatigue management in aviation.

This narrative review is divided into two sections. The first section describes the pathophysiology and epidemiology of fatigue and its possible causes, contributing factors and effects on aviation. The second section describes fatigue management (FM); preventive strategies and operational countermeasures. Figure 1 illustrates the structure and relationships between the various topics covered in this review. This review will conclude with a short discussion about the available evidence and possibilities for future research concerning FM in aviation.

## Definition of Fatigue

There are numerous definitions of fatigue, with varying classifications of the types of fatigue (e.g., mental and physical). Reduced physical performance has been shown to affect an individual’s ability to safely pilot an aircraft. For example, helicopter pilots show a significant deterioration of psychomotor performance in both hands and feet during sustained operations (McMahon and Newman, 2018). In the context of aviation, mental fatigue and sleepiness have been mentioned as the most important form of fatigue and will therefore be the focus of this review (Williamson et al., 2011). A recent review stressed the importance of distinguishing between sleepiness (i.e., drowsiness) and mental fatigue, emphasizing the differences in their causes and psychological and physical responses, while acknowledging that they interactively contribute to reduced performance and vigilance (Hu and Lodewijks, 2020). Sleepiness is mainly caused by circadian rhythm disruptions, sleep loss and time awake, whereas mental fatigue is mainly caused by time-on-task and cognitive workload (Balkin and Wesensten, 2011).

By contrast, the International Civil Aviation Organization (ICAO) definition of fatigue does not distinguish between mental fatigue and sleepiness: *“A physiological state of reduced mental or physical performance capability resulting from sleep loss, extended wakefulness, circadian phase, and/or workload (mental and/or physical activity) that can impair a person’s alertness and ability to perform safety related operational duties.”* (International Civil Aviation Organization (ICAO), 2020). This definition stresses the fact that fatigue is a multifactorial problem, with various causes and presentations, including impaired alertness and reduced performance, which may impair an individual’s abilities to perform his or her duties safely. Because the ICAO definition of fatigue is widely known and has been adopted by the aviation industry, this definition will be used throughout this review.

## Incidence of Fatigue in Flight

### Accidents and Incidents

A very illustrating example of fatigue and its effect on the safe piloting of an aircraft is provided by the, 2010 crash of Air India Express Flight 812, which crashed on landing in Mangalore, costing the life of 158 of the 166 persons aboard. Residual sleepiness and impaired judgement were believed to have contributed to this accident, as the cockpit voice recorder indicated that the captain had been asleep for the first 1h and 40min of the 2h and 5min flight (Court of Inquiry India, 2010). According to the National Transportation Safety Board (NTSB) this was the first instance of snoring recorded on a cockpit voice recorder.

Fatigue has also been identified in several other major aircraft accidents as either a cause or a contributing factor (National Transportation Safety Board, 2000, 2001, 2010; Libyan Civil Aviation Authority, 2013). In addition, aviation policies reflect the importance of fatigue in aviation safety. Since 1972, >200 safety recommendations issued by the NTSB focused on fatigue (Marcus and Rosekind, 2017). Moreover, pilot fatigue has been on the NTSB’s Most Wanted List of safety-related priorities since 1990 (Caldwell, 2012). These policies do not seem to have resulted in a significant improvement, however, as 23% of the major aviation accidents between 2001 and 2012 were attributed to fatigue, compared with 21% in a 1980 study (Lyman and Orlady, 1981; Marcus and Rosekind, 2017).

Similar to civil air operations, fatigue plays a major role in military aviation accidents. Fatigue was reported to be a causative factor in 12% of the US Navy’s Class A (most severe) accidents and in 25% of the US Air Force’s (USAF) night tactical fighter Class A accidents (Ramsey and McGlohn, 1997). A recent review of nearly 15years of USAF mishap reports showed that approximately 4% of all mishaps were fatigue-related, resulting in 32 fatalities and costing >$2 billion (Gaines et al., 2020). Interestingly, the percentage of fatigue-related class A mishaps was significantly higher at 24%, which is comparable to the 23% found in civil aviation (Marcus and Rosekind, 2017; Gaines et al., 2020). This higher percentage of Class A mishaps related to fatigue, may have been due to a more thorough investigation of human factors in the more severe mishaps.

### Prevalence of Fatigue

Fortunately, not every instance of pilot fatigue leads to an incident or accident, due to safety procedures in-place. Fatigue in-flight has been reported by 68–91% of commercial airline pilots (Jackson and Earl, 2006; Reis et al., 2016; Aljurf et al., 2018). Similar numbers are seen in the military, with 72% of military aviators admitting that they had flown at least once when they were so drowsy they could have easily fallen asleep. Moreover, a survey of USAF pilots and navigators found that 94% had experienced performance degrading effects of fatigue (Caldwell and Gilreath, 2002; Miller and Melfi, 2006). Because cabin crew members seem to experience less fatigue than pilots, and fatigue in cabin crew does not directly impair flight safety, this review will focus on fatigue in pilots (Houston et al., 2012).

## Causes of Fatigue

There are numerous factors in daily life which are associated with fatigue, like diet, exercise and physical fitness. In order to limit the scope of this section, we will only explore the causes of fatigue listed in the ICAO definition of fatigue (International Civil Aviation Organization (ICAO), 2020).

### Sleep Loss

The optimal duration of sleep per night varies among individuals, but 7–8h of sleep is recommended for adults (Hirshkowitz et al., 2015). The amount of sleep during the previous 24h was shown to be an independent predictor of threat and error management in a high-fidelity Boeing 747–400 simulator, as well as being a significant predictor of self-rated fatigue and mean response speed after international flight sectors (Petrilli et al., 2006; Gander and Signal, 2008). Also, restricted sleep, defined as <5h, during the previous 24h was associated with confusion (Drury et al., 2012). Sleep loss may be acute (not sleeping at all for an extended period of time, also known as sleep deprivation) or chronic “trimming” of sleep at night by 1 or 2h, also known as sleep restriction; (Goel et al., 2013; International Civil Aviation Organization (ICAO), 2020).

As many as 80% of army aviators and 90% of aviators in training were found to sleep <8h per night with these individuals sleeping an average of 6.6h per night (Tvaryanas and Thompson, 2006; Kelley et al., 2018; Bernhardt et al., 2019). Sleep decreased even more during operations, with average sleep per night being 6h prior to an operation and only 5.6h during an operation (Belland and Bissell, 1994). Similar patterns have been observed in civil aviation, with one study showing that 22% of Gulf Cooperation Council commercial airline pilots slept <6h per night (Aljurf et al., 2018). Another study reported that mean sleep duration decreased from 7.8h per night to <6h over a 7day duty period, leading to a cumulative sleep loss of 15h (Samel et al., 2004). Research has shown that the effects of sleep restriction accumulate, leading to a progressive reduction in performance, which intensifies as sleep restriction per night increases (Samel et al., 2004; International Civil Aviation Organization (ICAO), 2020).

In addition to reductions in hours slept, the quality of sleep may also be disturbed. For example, sleep at layovers may be complicated by transient factors such as unfamiliar or uncomfortable sleep environments, circadian disruptions, or situational stress (Caldwell, 1997). This can also be seen on military deployments; only 26.3% reported that sleep in the field was good, compared with 64.5% at home (Kelly and Efthymiou, 2019). These disturbances in turn may lead to a decrease in sleep quantity. For example, assessment of a rotary wing element deployed in Iraq found that 62.5% of the crew reported difficulties falling asleep and 48% reported difficulties staying asleep (Rabinowitz et al., 2009).

### Extended Wakefulness

Drive for sleep is associated with length of wakefulness (International Civil Aviation Organization (ICAO), 2020). This is due to a homeostatic process, in which an increase in time awake is followed by an increase in sleep pressure (Goel et al., 2013). This process results in sleepiness and a need for sleep when the sleep pressure increases above a certain threshold, and wakefulness when the sleep pressure decreases below a different threshold. A study by the NTSB showed that crews with a longer time since awakening (TSA; 13.8h for captains and 13.4h for first officers) made 40% more errors than crews with a shorter TSA (5.3h for captains and 5.2h for first officers; National Transportation Safety Board, 1994). Most of these errors were errors of omission, but crews with a longer TSA also made more procedural errors and tactical decision errors.

### Circadian Phase

The circadian body-clock is a neural pacemaker in the brain that monitors day/night cycle through ocular light input and determines the preference for sleeping at night (International Air Transport Association (IATA) et al., 2015). This clock controls the so-called circadian process, basically setting the thresholds for sleep pressure as described in the previous paragraph (Goel et al., 2013). Several periods during the day/night cycle are worth mentioning: The period during the circadian cycle when fatigue and sleepiness are greatest and people are least able to perform mental or physical work is called the window of circadian low (WOCL; International Air Transport Association (IATA) et al., 2015). This period, when the levels of attention are lowest, usually occurs between 2 and 6AM, but there are interindividual differences in timing (Valdez, 2019). Another well-known period is the post-lunch dip; occurring between 2 and 4PM when attention levels and the threshold for sleep are again low (Valdez, 2019). This dip is followed by a period with high levels of alertness occurring between 4 and 8PM, and then by the “evening wake maintenance zone,” the couple of hours just before one’s habitual bedtime, when it is very difficult to fall asleep (Goel et al., 2013; Valdez, 2019; International Civil Aviation Organization (ICAO), 2020).

Circadian rhythm can be disrupted by working at night (i.e., shift work) as this shifts the sleep/wake pattern and by time-zone transitions which cause sudden shifts in the day/night cycle, also called jet lag (International Air Transport Association (IATA) et al., 2015). These circadian rhythm disruptions can have a dual effect on cockpit performance. First, they can reduce (cognitive) performance and alertness when flying, such as during the WOCL, and second, they can lead to impaired sleep by displacement of sleep to the daytime when sleep quantity and quality are restricted (Gander et al., 1998b).

### Workload

The ICAO describes workload as “mental or physical” activity and identifies three aspects of workload: the nature and amount of work to be done; time constraints; and factors related to the performance capacity of an individual (International Civil Aviation Organization (ICAO), 2020). Both high and low workload situation may lead to reductions in performance, classified as active and passive fatigue, respectively (Hu and Lodewijks, 2020). High workload situations may exceed the capacity of the fatigued individual due to the high mental effort demanded, whereas low workload situations may lack sufficient stimulation which may unmask underlying sleepiness (Hu and Lodewijks, 2020; International Civil Aviation Organization (ICAO), 2020). The consequences of high and low workload situations may differ: low workload more commonly leads to less motivation and lower task engagement, whereas high workload leads to more distress and may impair sleep after work, due to the need to “wind down” (Hu and Lodewijks, 2020; International Civil Aviation Organization (ICAO), 2020).

## Effects of Fatigue

This section explores the effects on pilots of decreased mental performance, including impaired alertness.

### Impaired Alertness

Sleepiness and unintentionally falling asleep are possibly the two effects which are described most often by pilots when asked about their experience with fatigue in-flight. Fatigued individuals become less alert and may subsequently start to feel sleepy or drowsy. If these individuals do not get the rest period they need, the feeling of sleepiness may become overwhelming, resulting in the so-called micro-sleeps, defined as brief uncontrollable periods of sleep. This feeling of sleepiness decreases a pilot’s alertness, in ultimo resulting in unintentional (micro) sleeps, which leads to performance decrements (International Civil Aviation Organization (ICAO), 2020).

The numbers vary among different studies, but pilots consequently identify sleepiness and unintentional sleeps in-flight as effects of fatigue they have experienced. For example, 78% of Gulf Cooperation Council commercial airline pilots reported they had at least once felt so fatigued, they should not have been at the controls and 34% reported excessive daytime sleepiness (Aljurf et al., 2018). This is also seen in military aviation, with 72% of the Army pilots reporting that they had flown at times when they were so drowsy they could have easily fallen asleep (Caldwell and Gilreath, 2002). The percentage of pilots that had actually experienced unintentional sleep in-flight during their career was similar in both studies mentioned above; 45%(Caldwell and Gilreath, 2002; Aljurf et al., 2018). However, this percentage was only 21% in a different study on Army pilots, while merely 3% of the Air Force pilots and navigators reported they had never fallen asleep in-flight (Miller and Melfi, 2006; Kelley et al., 2018). This variation may be explained by the different populations being studied, with variations in hours flown, average duration of flights and planning of flights.

### Decreased Performance

The decrease in performance when fatigued was already demonstrated in the late nineteenth century after keeping subjects awake for almost 90h (Patrick and Gilbert, 1896). More recent studies showed that just 2h of sleep loss leads to performance decreases equal to those observed after consuming two to three bottles of beer (Roehrs et al., 2003). Impairment of cognitive functioning is particularly noticeable when measuring executive functioning, sustained attention and long-term memory (Lowe et al., 2017). Although sleep loss has similar effects on functional neuroimaging techniques among subjects, individual performance on cognitive measures are found to vary considerably. This may be due to trait-like (for example genetic) differential vulnerability among individuals, or by compensatory changes in neurologic systems involved in cognition (Goel et al., 2009).

Similar reductions in performance have been observed in fatigued pilots. Several studies showed flight performance significantly declines after 24h of wakefulness (Caldwell et al., 2004a; Previc et al., 2009). This was confirmed by a survey of pilots, with >80% reporting that fatigue affected their flight performance (Gregory et al., 2010). In two other studies 67 and 90% of pilots reported ever having made mistakes due to fatigue (Reis et al., 2013; Aljurf et al., 2018).

A study of USAF pilots and navigators found that 94% reported performance degrading effects of fatigue, which contributed to decreased situational awareness in 73%, slowed reaction time in 67% as well as increased distractibility (43%), forgetfulness (41%) and apathy (33%; Miller and Melfi, 2006). Reduced in-flight attention and lack of concentration were reported by 23 and 25% of the commercial pilots, respectively performing short-haul (SH) and long-haul (LH) operations, with 80% of a group of SH commercial pilots regarding their judgement as impaired while flying (Bourgeois-Bougrine et al., 2003; Jackson and Earl, 2006). Fatigue in pilots has also been shown to lead to an increase in heightened emotional activity, which in turn leads to impaired higher-order cognitive processing (Drury et al., 2012). Additionally, fatigue was found to lead to significant visual perceptual impairment and visual neglect, although instrument scanning was apparently unaffected (Russo et al., 2004, 2005; Previc et al., 2009). Other identified consequences of fatigue include a decrease in social communication, reaction time and cognitive flexibility and hand-eye coordination (Bourgeois-Bougrine et al., 2003; Petrilli et al., 2006; O’Hagan et al., 2018).

### Long-Term Health Effects

Despite not being included in the ICAO definition, research has shown that fatigue may cause long-term health effects. Although these effects may have a limited influence on the performance of a fatigued pilot, they may lead to long-term reductions in performance.

Fatigue has been shown to reduce working ability in general and may be associated with feelings of depression or anxiety (O’Hagan et al., 2016; Pasha and Stokes, 2018; Pellegrino and Marqueze, 2019). Moreover, severely fatigued pilots had higher rates of excessive daytime sleepiness, depression and obstructive sleep apnea than non-fatigued pilots (O’Hagan et al., 2018). Levels of cardiovascular strain were found to be higher on day 4 than on day 1 of a work period, consistent with the hypothesis that fatigue and work periods increase cardiac strain among aircrew (Goffeng et al., 2019a). These long-term detrimental effects of circadian rhythm disruption and fatigue have also been observed in other occupational areas. For example, poor sleep hygiene in the military has been associated with cardiovascular disease, substance abuse and mood disorders (Good et al., 2020). Moreover, the International Agency for Research on Cancer in 2019 classified shift work involving circadian disruption as a probable human carcinogen, with positive associations between night shift work and cancers of the breast, prostate, colon and rectum (Straif et al., 2007; International Agency for Research on Cancer (IARC), 2019). Epidemiologic studies, mainly including nurses, showed an association between sustained night work and a 50–100% higher incidence of breast cancer (Touitou et al., 2017). Moreover, habitually short sleep or circadian phase disruptions have been linked to conditions such as weight gain, obesity, diabetes, and hypertension, as well as to increased mortality (Goel et al., 2013).

## Contributing Factors in Aviation

Section “Causes of fatigue” focused on the causes of fatigue, based on the ICAO definition. This section will explore some important factors contributing to fatigue in the aviation industry, including the relevance of the type of operation, jet lag and planning aspects. Another important factor, although not specific to the aviation industry, is the inter-individual differences which will also be discussed in this section.

### Type of Operation

SH operations mostly consist of flights up to 3h in duration, whereas LH flights typically last for 6–12h. LH operations were thought to impose a higher risk of fatigue than SH operations, with manifestations of fatigue reported by 60% of LH pilots and 49% of SH pilots (Bourgeois-Bougrine et al., 2003). More recent studies, however, found that the prevalence of fatigue was significantly higher in SH than in LH operations (93% vs. 84.3%), with a 2.945 added risk of fatigue in SH pilots (Reis et al., 2013, 2016). This shift in prevalence may have been due to developments in air travel. Airlines continue to offer more flights over relatively short distances, resulting in SH pilots performing more take-offs and landings per duty period than LH pilots, with the former having a higher workload (Powell et al., 2007, 2008). Along with the differences in work schedules between SH and LH pilots, the factors causing fatigue in these individuals also seem to be slightly different. For example, fatigue in LH pilots was due mainly to disturbances in their circadian rhythm, resulting from night flights and jet lag, whereas fatigue in SH pilots was due to prolonged duty periods with a long time-on-task and sleep loss due to early awakening (Bourgeois-Bougrine et al., 2003). However, night-time flights by SH-pilots may also lead to circadian rhythm disruption.

### International Flights

The circadian body-clock is unable to adapt immediately to changes in time zones. This period of adaptation, or jet lag, generally lasts longer when travelling eastward, after crossing multiple time zones and when exposure to the local day/night cycle is limited (Gander et al., 2013a). Pilots not attuned to the local time zone, often find it difficult to recuperate from outbound flights. Pilots report difficulties getting enough sleep during the layover due to jet lag, with return flights associated with increased fatigue, measured both subjectively and objectively (Samel et al., 1997; Paul et al., 2001b; Eriksen et al., 2006). This difficulty of acclimatization to the local time zone is more prominent when comparing short (1–2days) and long (3–4days) layovers. Although pilots got more sleep per day during a short layover, they remained more fatigued and had poorer performance ratings than after a long layover (Roach et al., 2012a). When adaptation to the local time zone is inhibited by working night shifts at the layover destination, a circadian drift occurs, with the internal circadian clock adapting a cycle length of >24h (Gander et al., 1998a). This leads to shifts in sleep/wake patterns and consequent sleepiness (Gander et al., 2016).

### Planning Aspects

Work organization is frequently reported by pilots as being a factor affecting the incidence of fatigue. The rostered duty pattern was associated with 27% of all fatigue reports of a commercial airline, with 25% reporting that mission planning was a primary contributor to fatigue (Houston et al., 2012; Morris et al., 2018). Other aspects of work organization identified by pilots as factors contributing to fatigue are more consecutive working nights, longer career duration, more time on the night shift and no good place to sleep onboard (Pellegrino and Marqueze, 2019). Another parameter that has been of increasing interest is the crew composition, with fatigue being a greater risk for two-member than for three-member flight crews, probably due to two-member crews having diminished opportunities of sleeping in-flight (Eriksen et al., 2006).

Assessments of the effects of Flight Duty Period (FDP) on fatigue have identified several subfactors, including duty length, time of day and number of flight segments (Bourgeois-Bougrine et al., 2003; Powell et al., 2007, 2008; Vejvoda et al., 2014). However, the relative importance of these factors has differed among studies.

#### Duty Length

In addition to the length of actual time in the air, the entire duty length (flight time plus time needed for briefings, administration, etc.) is an important cause of fatigue (Caldwell et al., 2009). This association was described after Operation Desert Shield (Bisson et al., 1993). Since then, many studies have confirmed that duty length is a major predictor of fatigue with some studies reporting it as the most important contributor to fatigue (Bourgeois-Bougrine et al., 2003; Powell et al., 2007; Reis et al., 2016; Morris et al., 2018).

#### Time of Day

The start time of the FDP can affect the amount of sleep that pilots are able to get the previous night, with early start time being associated with significantly less sleep the night before. For example, sleep time has been reported to average 6.5h when take-off was at 8AM and 5.5h when duty started between 4 and 5AM (Paul et al., 2001b; Roach et al., 2012b). Shorter sleep time was associated with elevated levels of fatigue and accidents, with >50% of the USAF fatigue-related mishaps occurring between 1 and 7AM (Reis et al., 2016; Gaines et al., 2020).

However, recent studies have shown that FDPs ending late at night or in the early morning hours may lead to an increased level of fatigue. Pilots were significantly more fatigued during duty and after duty periods starting later in the day, despite their prior sleep being longer and their duty time shorter (Vejvoda et al., 2014). This may be due to an extended period of wakefulness or to flying during the WOCL, which has been shown to lead to higher levels of fatigue (Powell et al., 2008). Other parameters were similarly affected, with reaction time being slower on flights arriving at 6–9AM than later, and evening crews spending more time with so-called “heavy eyelids,” i.e., experiencing the urge to rest your eyes (Eriksen and Akerstedt, 2006; Gander et al., 2014a). SH and LH FDPs covering the whole domicile night (12–6AM at home base) were most consistently associated with reduced sleep–wake ratio (33%) and subjective alertness (50%; Sallinen et al., 2017).

#### Number of Flight Segments

Flying a greater number of segments was found to contribute significantly to a higher level of fatigue at the end of the last duty sector (Powell et al., 2007, 2008; Reis et al., 2016). For example, fatigue ratings on the Psychomotor Vigilance Test (PVT) were significantly higher for a 5-segment than for a 1-segment duty day, although simulator flight performance of both was similar (Honn et al., 2016). Additionally, reaction time was increased significantly as the number of flight segments during the duty period increased, although precision remained unchanged (Goffeng et al., 2019b). This increase in fatigue ratings may be attributed to an increased workload caused by the extra take-offs and landings per FDP.

### Inter-Individual Differences

Even when taking into account all of the abovementioned factors, there are large inter-individual differences in the levels of fatigue experienced by pilots and their capability to cope with fatigue (Petrie et al., 2004). This has been shown in military aviation, even among highly-trained individuals such as fighter-jet pilots with up to 66% of the variations in performance under sleep-deprivation condition being attributed to inter-individual differences (Van Dongen et al., 2006). For example, pilot age is an important factor, as a higher age is associated with lesser sleep quality, increased sleep loss and a decreased speed of adaptation to crossing time zones (Graeber et al., 1986; Gander et al., 1993; Gander and Signal, 2008; van Drongelen et al., 2017). Circadian type may also be related to the incidence of fatigue, although its extent is unclear (Van Dongen et al., 2006). One study reported that being an evening type was associated with easier adaptation to time-zone changes; another identified evening type as a risk factor for fatigue; and a third study suggested that morning types may be more likely to utilize fatigue mitigation strategies when concerned about fatigue (Gander et al., 1993; van Drongelen et al., 2017; Morris et al., 2020). Other factors that may negatively affect the incidence of fatigue include disturbances in work-life balance, limited physical activity, increased duration of shift work and impaired domestic relationships (Tvaryanas and MacPherson, 2009; van Drongelen et al., 2017). Gender, however, was not associated with fatigue (Caldwell and LeDuc, 1998).

## Fatigue Management

Fatigue Management (FM) refers to the methods by which aviation service providers and operational personnel address the safety implications of fatigue (International Civil Aviation Organization (ICAO), 2011). In 1933 the Aero Medical Association of the US adopted a resolution to lower the maximum number of flying hours per month from 110 to 85, basically introducing the first FTL (Rothert et al., 1988). These FTL are the first step of the so-called “prescriptive approach” to FM (International Civil Aviation Organization (ICAO), 2011). This approach is called “Fatigue Management” in EASA regulations, whereas the US Federal Aviation Administration (FAA) uses the term “Fatigue Risk Management Program.” The second step in this approach is the management of fatigue-related risks by the service provider (e.g., airline operator) in their Safety Management System.

These set limits address the predicted risks of fatigue in general. However, this merely resembles reality and consequently these limits and this approach have been subject to criticism. Critics have pointed out the considerable variations among regulations, the restrictive nature of the limitations and the regulations’ lack of a basis in modern fatigue science (Dawson and McCulloch, 2005; Caldwell et al., 2009; Missoni et al., 2009). EASA countered some of this criticism by tasking an expert panel to perform a scientific and medical evaluation of some of the FTL, especially focusing on the best options to mitigate fatigue and improve flight safety (MOEBUS Aviation, 2008). However, the adherence of FTLs to strict boundaries, even when the risk of fatigue is identified as a gradient function remains an important drawback of this prescriptive approach to FM (Romig and Klemets, 2009). Subsequently, several countries, such as New Zealand, Australia and Canada, have started to implement less prescriptive approaches to FM. These new approaches have allowed service providers to propose FTLs specific to their companies or operations for approval (Signal et al., 2008). This “performance-based approach” uses a Fatigue Risk Management System (FRMS), called Fatigue Risk Management by the EASA, that focuses on managing the actual risk of fatigue in specific operations. Thus, rather than strict adherence to the FTL, these providers are allowed more flexibility while ensuring that their fatigue risks are managed at least as well as under the prescriptive approach. In 2011, the ICAO and the IATA published the FRMS Implementation Guide for Operators, for the first time allowing the implementation of FRMS, using the following definition (International Air Transport Association (IATA) et al., 2011): *“a data-driven means of continuously monitoring and managing fatigue-related safety risks, based upon scientific principles, knowledge and operational experience that aims to ensure relevant personnel are performing at adequate levels of alertness.”*(International Civil Aviation Organization (ICAO), 2011). For approval of an alternative FTL, an airline must estimate the level of fatigue-related risks associated with the operation(s), propose appropriate mitigations to manage those risks, and continuously monitor fatigue and related risks (Wu et al., 2018). Fatigue and sleep behaviour modelling tools are the cornerstone for these estimations of fatigue-related risks, predicting the degree of fatigue caused by a stated work-rest cycle. Because these are population-based tools set for average individuals, they must be optimized and validated for targeted populations (Paul et al., 2020). The requirement to monitor fatigue and its related risks is one of the largest disadvantages of this system, which has been described by some as a bureaucratic process that provides an illusion of fatigue risk control (Bourgeois-Bougrine, 2020). Consequently, the implementation of an FRMS is quite demanding for the service provider, making the “prescriptive approach” with set FTL the best solution in some situations. Despite this, FRMS is currently considered the future of FM and has been implemented in many international regulations as an alternative to prescriptive FTL, with updated versions of both the IATA Guide and ICAO Manual (Federal Aviation Administration (FAA), 2013; European Union Aviation Safety Agency (EASA), 2014; International Air Transport Association (IATA) et al., 2015; International Civil Aviation Organization (ICAO), 2020).

Despite their differences, there are many similarities between these two approaches to FM. First, FM is always a shared responsibility between States, Service Providers and individuals (International Civil Aviation Organization (ICAO), 2020). Both approaches must be based on scientific knowledge, as well as operational experience, and should take into account the need for adequate sleep, circadian rhythms, the effects of fatigue and workload on performance, and the operational context. FM and its measures to decrease the impact of fatigue in flight operations can be divided into two categories: preventive strategies and operational countermeasures (Gander et al., 1998b). The former entails procedures to ensure crew members get optimal sleep during layovers and before duty, whereas the latter includes actions that can be taken in-flight to maintain alertness and performance.

## Preventive Strategies

### Scheduling

Historically, the FTL started in 1934 with a maximum allowance of 85 flight hours per month, also known as ‘Decision 83’ (Rothert et al., 1988). However, as described in section “Planning aspects”, both the number of flight hours and the scheduling of the FDP are important in managing the risks of fatigue. Current EASA and FAA regulations state that the maximal duration of the daily FDP depends on the start time and number of scheduled flights per FDP (Federal Aviation Administration (FAA), 2012; European Union Aviation Safety Agency (EASA), 2014). The latest regulations of EASA and FAA seem to be in greater agreement, although they still vary, for example in allowed length of FDPs.

A recent study shows that the strain of night-time flights is still not fully reflected in EASA regulations, stressing the importance of sufficient sleep before night duties (European Union Aviation Safety Agency (EASA), 2019). Another study found that the prescriptive rules were insufficient to mitigate the risk of fatigue during high season months (Rodrigues et al., 2020).

The introduction of FTL into military aviation is complicated by the wide variety of aircraft, missions and crew configurations. The NATO standard “Fatigue Management in Air Operations” stresses the responsibilities of each country to regulate maximum flying and operating times and to provide compulsory rest periods, while determining the maximum allowed accumulated flying hours (North Atlantic Treaty Organization (NATO), 2020). The Military Aviation Requirements of the RNLAF do not specify the FTL, except for the maximum accumulated flying hours, but FTLs are described in operations manuals for various aircraft, allowing for differences in FTLs among aircraft types (Military Aviation Authority, 2020). Additionally, operational necessity might call for extension of the allowed duty period at the commander’s approval. These differences and the possibility of deviations make the implementation of additional fatigue countermeasures paramount.

### Sleep Hygiene

Although sufficient and good quality sleep is the best prevention for fatigue, most chronic sleep difficulties in otherwise healthy individuals are likely due to poor sleep hygiene (Caldwell, 1997). Individuals may have difficulties maintaining proper sleep hygiene while coping with irregular flight schedules and circadian disruption (van Drongelen et al., 2014). Therefore training crew members about fatigue and sleep hygiene is of utmost importance and is currently regarded as a mandatory aspect of FM (European Union Aviation Safety Agency (EASA), 2014; International Civil Aviation Organization (ICAO), 2020). Implementation of these types of training was shown to reduce self-reported fatigue and to improve sleep quantity and performance (Rosekind et al., 2006; van Drongelen et al., 2014). Inadequate sleep hygiene and environmental factors preventing sleep are still reported and remain areas for improvement (Zakariassen et al., 2019). In addition, getting optimal amounts of good quality sleep is especially difficult for persons on military deployment (Kelly and Efthymiou, 2019).

### Pre-flight Naps

As described in section “Effects Of Fatigue”, a longer period of wakefulness leads to a higher sleep pressure, which causes more fatigue. People who are well rested and have an in-sync sleep–wake cycle can maintain high levels of alertness and performance for approximately 16h (Dijk et al., 1992; Van Dongen et al., 2003). Daytime naps, before shift work decrease fatigue and increase performance, as do naps during the night shift (Garbarino et al., 2004; Brooks and Lack, 2006; Caldwell et al., 2009; Ruggiero and Redeker, 2014; Martin-Gill et al., 2018). Both daytime and night-time naps are commonly used in military and commercial aviation (Petrie et al., 2004; Gregory et al., 2010; Kelly and Efthymiou, 2019; Zakariassen et al., 2019). Pilots aged >50yrs. are more inclined to use this strategy (Petrie et al., 2004). Text Box 1 describes aspects to take into consideration when napping.

***Text Box 1**Aspects to take into consideration when napping*.

*Generally, a longer nap leads to a longer beneficial effect*(*Driskell and Mullen, 2005*; *Mulrine et al., 2012**) However, naps as short as 10 min have been shown to reduce sleepiness and fatigue and to improve cognitive performance (**Brooks and Lack, 2006*). Nap timing is as important as nap duration, with naps of the same length having different effects at different times of day (Fatigue Countermeasures Working Group, 2018*). Although naps taken later in the night (near the WOCL) lead to more efficient sleep, they do not lead to better performance, which can be attributed to increased sleep inertia when awakening during the WOCL (**Kubo et al., 2007*; *Caldwell et al., 2009*; *Hilditch and McHill, 2019**)*.

*Sleep inertia is the transitional state of lowered arousal immediately after awakening, with the most significant reductions in performance and alertness occurring during the first 30 min after awakening (**Tassi and Muzet, 2000*; *Hilditch and McHill, 2019**). Sleep inertia may be equal to 40 h of sleep deprivation in impairing performance and the effect of sleep inertia may be exacerbated by awakening after sleep loss or from deeper stages of sleep (**Hilditch and McHill, 2019**). Limiting the duration of the nap does not necessarily prevent deeper stages of sleep, and the relationship between deeper stages of sleep and the occurrence of sleep inertia remains inconsistent in people taking short naps (**Hilditch et al.,2016*, *2017a*,*b**). Therefore, a sleep inertia recovery period is needed after every nap, regardless of the length of the nap (**Hilditch et al., 2020**). Caffeine may reduce the impact of sleep inertia, as may light, sound and temperature, although additional research is needed (**Hilditch and McHill, 2019**)*.

### Sleep During Layovers

Sleep obtained during layovers enables recovery from the outbound flight and preparation for the next flight (Roach et al., 2012a). Factors such as layover duration, flight direction, number of time zones crossed and scheduling affect the positive gain from sleep during layover (Kandelaars et al., 2006; Cosgrave et al., 2018). Fatigue levels seem to remain higher and performance levels more impaired relative to baseline after a short than after a long layover (Roach et al., 2012a). Total time slept in the 24h prior to the inbound flight was highest when the layover included a full domicile night-time period, indicating that circadian end-time of the layover was a key determinant of fatigue at duty start time (Cosgrave et al., 2018). Additionally, social cues may lead to a start of acclimation to local timing during layovers, leading to shifts in sleeping patterns (Kandelaars et al., 2006; Lamp et al., 2019).

### Hypnotics

Demanding schedules, especially during military missions where sleep quality is often decreased due to ambient factors (e.g., noise, heat and sleeping during the day), may make it difficult to obtain sufficient sleep. Under such conditions, several organizations, predominantly military, allow the use of hypnotics to optimize the quality of crew rest. Temazepam, zolpidem and zaleplon are frequently prescribed hypnotics in aviation. Table 1 provides an overview of these hypnotics and their characteristics. The hypnotic prescribed to achieve optimal results depends on circumstances, such as time of day, length of the sleep period and the probability of an earlier-than-expected awakening. Briefly: temazepam is optimal for maintaining sleep or optimizing sleep during the day, whereas zolpidem and zaleplon are better for initiating sleep and short naps. Additionally, the effectiveness and occurrence of side effects may differ between individuals and pilots may have their own preference. These factors can be assessed during ground testing, whereafter the flight surgeon and pilot together can decide on the best hypnotic for him or her.

**Table 1:** Pharmaceutical countermeasures.

Pharmaceutical	Features	Dosage	Side effects	Indications/ Clinical benefits	Contraindications
**Hypnotics**					
Temazepam *Benzodiazepine*	#GABA agonist	7.5–30mg	#Common: hypotension, somnolence, headaches, blurred vision	#Facilitating and improving day- and night-time sleep	< 8h available for sleep
	T_max_ 50min				
	T_1/2_ 8–12h				
					#Sleep apnoea, pulmonary disease, pregnancy
			#Rare: apnoea, movement and memory disorders		
				#Treating sleep-maintaining difficulties in westward travellers	
			#Dependency and tolerance may occur when used for a longer period		
Zolpidem *Non-benzodiazepine-based hypnotic*	#Selective agonist of benzodiazepine-1 receptor	5–10mg (immediate release formulation)Women: 5mg due to slower elimination	#Common: diarrhoea, nausea, drowsiness, headaches	#Facilitating naps of moderate (4–7h) duration, especially outside optimal nap times	< 4–6h available for sleep
					#Sleep apnoea, myasthenia gravis, psychiatric
	T_max_ 0.5–3h				
	T_1/2_ 2.5h				

					#History of complex sleep behaviours
			#Rare: agitation, confusion, complex sleep behaviours		
			#When used for a longer period dependency and tolerance may occur	#Treating sleep-onset difficulties in eastward travellers	
Zaleplon *Non-benzodiazepine-based hypnotic*	#Selective agonist of alpha subunit of the GABA_A_ omega-1 receptor	5–10mg	#Common: headaches	#Facilitating naps of short (1–4h) duration, especially outside optimal nap times	< 3h available for sleep
			#Rare: Nausea, dizziness drowsiness, amnesia, psychiatric symptoms eye pain, complex sleep behaviours		#Sleep apnoea, myasthenia gravis
					#History of complex sleep behaviours
	T_max_ 1h				
	T_1/2_ 1h				
				#Treating sleep-onset difficulties in eastward travellers	
			#When used for a longer period dependency and tolerance may occur		
				#Improving the initiation of early	
				night-time sleep	
Melatonin *Chronobiotic and weak hypnotic*	#Melatonin receptor agonist	0.1–10mg	#Common: Headaches, somnolence	#Improving day- and night-time sleep	#Administration of melatonin at wrong time can shift circadian rhythm in wrong direction, worsening symptoms of fatigue.
	T_max_ 50min		#Rare: Nausea, hypertension, dizziness, agitation, amnesia		
				#Increasing circadian adaptation in long-haul crew	
	T_1/2_ 45min				
				#Improving the initiation of early night-time sleep	
**Stimulants**					
Caffeine	#Adenosine receptor antagonist	200–300mg, may be repeated	#Dose-dependent: agitation, irritability, tremor, dysrhythmia and gastrointestinal complaints	#Short-term improvement of alertness and performance after sleep deprivation, when a limited level of medical oversight is available.	#Hypertension, hyperthyroidism, epilepsy, mania, schizophrenia and ulcers
	T_max_ 30–120min				
	T_1/2_ 4–6h				
			#Daily (higher) usage may lead to tolerance and withdrawal symptoms		
Dextroamphetamine	#Modulators of the dopaminergic system	10mg, may be repeated every 4h	#Common: tachycardia, hypertension, abdominal complaints, headaches, jitteriness	#Longer duration of improvement of attention, flight performance and decrease feelings of fatigue	#Symptomatic cardiovascular disease, moderate to severe hypertension, hyperthyroidism, glaucoma and psychotic disorders
	T_max_ 1.5h				
	T_1/2_ 10h				
			#Dose-dependent: euphoria, tunnel vision, motor restlessness		
				#Only under proper medical supervision	
			#Recovery sleep may be less restful		
			#Significant potential for addiction and abuse		
Modafinil	#Modulator of the GABAergic and glutamatergic neurotransmission system	100–200mg, may be repeated every 4–5h	#Common: headaches, tachycardia, diarrhoea, insomnia, anxiety, dizziness, visual impairments	#Improvement of attention, cognition, flight performance and mood	#Hypertension and arrythmias
	T_max_ 2–4h				
	T_1/2_ 12–15h		#Rare: hypertension, agitation, arrythmias		
			#Recovery sleep may be less restful		

#### Temazepam

Temazepam, a benzodiazepine, exerts its effects by increasing the activity of the ƴ-aminobutyric acid (GABA) system, inducing its inhibitory action on the central nervous system. By doing so, benzodiazepines induce sedative, anxiolytic, hypnotic, muscle relaxant and anticonvulsant effects (Madari et al., 2021). They cause side effects such as movement and memory disorders, as well as dizziness, lethargy and apnoea (Relative) contraindications include sleep apnoea, pulmonary disease, liver disease and temazepam is not to be used during pregnancy. Furthermore, when used for a longer period of time, benzodiazepine use is associated with dependency, tolerance and withdrawal (Toyoshima et al., 2021). Hypotension, somnolence, headaches and blurred vision are common adverse effects especially associated with temazepam use (Madari et al., 2021).

The recommended dosage of temazepam is between 7.5 and 30mg, to be taken directly before bedtime. Because of its relatively long half-life of 8–12h, temazepam should be given to individuals who have at least 8h available for sleep, with low likelihood of sleep interruption after drug administration. Temazepam has been shown to facilitate and improve night-time sleep without leading to residual effects in the morning (Mattila et al., 1984; Wesnes and Warburton, 1986). It is also effective for improving daytime sleep (Wesnes and Warburton, 1986; Bricknell, 1991; Caldwell et al., 2003b). These qualities make it a good choice for facilitating night-time sleep after westward travel, inasmuch as an individual’s body-clock essentially perceives that it is daytime during the local night (Donaldson and Kennaway, 1991). To prevent drug tolerance or dependence, temazepam should be used for less than 3 to 7 consecutive days (Caldwell et al., 2009). Temazepam at a dose of 10mg is one of the 2 possible hypnotics prescribed to members of the RNLAF, for a maximum of 7 consecutive days. After administration, the aircrew is grounded for 12h (Military Aviation Authority, 2021).

#### Zolpidem

Zolpidem was one of the first non-benzodiazepine-based hypnotics, also known as Z-drugs, to become available. Zolpidem selectively binds to the benzodiazepine-1 receptor, inhibiting neuronal excitability and action potentials (Madari et al., 2021). More common side effects of zolpidem are diarrhoea, nausea, headaches, dizziness, drowsiness and the worsening of mood issues. Other less common adverse effects include agitation, confusion, allergic reactions and visual disturbances. A recent study showed that only 3.5% of aircrew members experienced side effects during a ground test, with next-day drowsiness and headaches being the side effects most often reported (Sen Kew and See, 2018).

Z-drugs, and zolpidem in particular, are more often associated with complex sleep behaviours (e.g., parasomnias) then benzodiazepines (Harbourt et al., 2020). This may be explained by the maintenance of slow-wave sleep combined with the decreased muscle-relaxing effect of Z-drugs compared to benzodiazepines (Chopra et al., 2013). Even though these complex sleep behaviours are a rare side effect of zolpidem, they may lead to injury and therefore physicians are strongly advised to screen for and warn users about this adverse effect (Earl and Van Tyle, 2020). Other (relative) contraindications include obstructive sleep apnoea, myasthenia gravis, severe liver disease and respiratory depression. Furthermore, the use of Z-drugs is more often associated with the occurrence of psychiatric symptoms and cortical dysfunction (Toyoshima et al., 2021).

Due to their more distinct pharmacological profile and shorter half-life compared to benzodiazepines, it was hypothesized that rebound insomnia, tolerance and dependency would occur less when using Z-drugs. This, unfortunately does not seem to be the case, as the prevalence of dependency and tolerance seems to be similar (Schifano et al., 2019; Curado et al., 2021).

The recommended dosage of zolpidem immediate release formulation is 5–10mg, to be taken immediately prior to sleep initiation (Madari et al., 2021). As women are shown to have a slower elimination of zolpidem, the recommended dosage is5 mg for women (Center for Drug Evaluation and Research, 2013). Zolpidem has been shown to be efficient in promoting night-time as well as daytime sleep without performance deficits upon awakening (Sicard et al., 1993; Holm and Goa, 2000; Batejat et al., 2006). Zolpidem has a half-life of 2.5h and it reaches its peak plasma concentration after 0.5–3h. Awakening 1.5h after administration of 10mg zolpidem was shown to lead to significant cognitive performance decrements compared to placebo (Dinges et al., 2019). These were not found after administration of 5mg zolpidem, or when awakening 6.7h after administration. In order to avoid cognitive performance decrements after awakening individuals taking zolpidem (especially when using 10mg) should have at least 4–6h available for sleep after its administration.

Zolpidem is considered a good choice for facilitating naps of moderate durations (4–7h), especially outside of optimal nap times, as well as treating sleep-onset difficulties in eastward travellers (Caldwell et al., 2009). Zolpidem at a dose of 5mg is the second hypnotic available to RNLAF aircrew, followed by a grounding period of 6h (Military Aviation Authority, 2021).

#### Zaleplon

Zaleplon is another Z-drug and is thought to exert its action by selectively binding to the alpha subunit of the GABA_A_ omega-1 receptor (Madari et al., 2021). Headaches are the most common side effect of zaleplon. Other less common side effects include abdominal pain, nausea, dizziness, drowsiness, amnesia, eye pain and allergic reactions. Like the other Z-drugs, its use is also associated with the occurrence of complex sleep behaviours, psychiatric symptoms and cortical dysfunction (Toyoshima et al., 2021; Relative) contraindications include obstructive sleep apnoea, myasthenia gravis, severe liver or renal disease and respiratory insufficiency.

The recommended dosage of zaleplon is 5–10mg, to be administered just before bedtime or during middle of the night awakenings (Madari et al., 2021). It’s half-life of 1h is shorter than that of zolpidem, making it even less likely to lead to residual drug effects. When awakened at T_max_, 1h after 10mg zaleplon administration, very few negative effects on cognitive performance were found and none were found after awaking in the morning (after 6.7h; Dinges et al., 2019). This makes it a good choice for initiating very short daytime naps (1–4h). In order to avoid any cognitive decrements, individuals taking zaleplon should not perform activities within 3h of its ingestion (Paul et al., 2004). Another positive effect of zaleplon is its improvement of initiation of early night-time sleep, which may benefit personnel who have to report for duty early in the morning (Fry et al., 2000). Similarly, zaleplon may be useful in treating sleep-onset insomnia in eastward travellers with mild cases of jet lag (Caldwell et al., 2009). Zaleplon is not registered in the Netherlands and therefore not used by the RNLAF.

### Chronobiotics

Melatonin is a hormone produced by the pineal gland which regulates the sleep–wake cycle (Madari et al., 2021). Dosages from 0.1 up to 10mg have been described. Melatonin administration can shift circadian rhythms in humans, delaying circadian rhythm when administered in the morning and advancing circadian rhythm when administered in the afternoon (Lewy et al., 1992). Furthermore, it also works as a weak hypnotic, improving sleep onset latency and total sleep time, even though this effects are smaller than those of benzodiazepines or Z-drugs (Madari et al., 2021). Melatonin at a dosage of 10mg has been shown to facilitate early circadian sleep in aircrew (Paul et al., 2001a). When taking after a night shift, it may increase sleep length during daytime sleep and night-time sleep (Liira et al., 2014). In theory this is useful, especially for LH crew members who require a more rapid circadian adaptation when back at home or SH crew members having early morning take-offs, thereby advancing sleep in the early evening. Possible side effects are minor, e.g., headaches and somnolence (Madari et al., 2021). However, administration of melatonin at the wrong time can shift the circadian rhythm in the wrong direction, resulting in subsequent sleepiness and decreased performance. For this reason, the use of melatonin is prohibited by most aviation authorities (Caldwell et al., 2009; Simons and Valk, 2009). The RNLAF allows the use of melatonin at dosages of up to 0.5mg only when prescribed by a flight surgeon (Military Aviation Authority, 2021).

## Operational Countermeasures

### In-flight Napping

Different types of in-flight sleep have been described for pilots. Unplanned and involuntary sleeping on the flight deck is either unintentional napping or microsleeping (i.e., episodes of sleep lasting a fraction of a second to several seconds). Controlled rest (CR), defined as an intentional in-seat nap on the flight deck, is widely used and endorsed by both the ICAO and EASA, as long as it remains within the constraints of applicable policy (European Union Aviation Safety Agency (EASA), 2012; Hartzler, 2014; Fatigue Countermeasures Working Group, 2018; International Civil Aviation Organization (ICAO), 2020). CR differs from bunk rest (BR), during which a pilot leaves the flight deck to rest in a designated crew rest facility. Other differences between CR and BR are length and planning; CR consists of unscheduled short naps, whereas BR is scheduled and often lasts at least 1h. Further, BR is exclusive to augmented crews, consisting of more than two pilots (International Air Transport Association (IATA) et al., 2015). Despite these distinct differences, CR and BR have similarities both in their effects and in the aspects to take into consideration (see Text Box 1). Napping not only helps to restore alertness and performance, but also reduces sleep pressure and feelings of fatigue (Hartzler, 2014). These differences and effects are summarized in Table 2.

**Table 2:** Comparison between controlled rest and bunk rest.

	Controlled rest	Bunk Rest
Duration	40–45min	> 1h
Place	In-seat on the flight deck	Designated crew rest facility
Planning	Unscheduled	Scheduled
Indication	Unexpected fatigue due to sleepiness in-flight	Scheduled naps on longer flights
Type of operations	Any, but typically used on shorter (<10h) night flights	Longer flights, especially ultra-long-range operations
Preconditions	#Minimal 20min sleep inertia recovery period after nap	#Sleep inertia recovery period after nap
		#Augmented crew
	#Waking pilot has sufficient situational awareness, implement pre-nap briefing	
Factors affecting quantity and quality of nap	#Environmental factors (e.g., seat reclination)	#Duration of the nap
		#Scheduling (e.g., consider circadian timing of nap and previous amount of sleep) # Environmental factors (e.g., noise and comfort)
		#Psychological factors (e.g., provide pilots with relaxation training)
Effects	#Restore alertness and (cognitive) performance	
	#Reduce sleep pressure and feelings of fatigue	

#### Controlled Rest

CR is most often used during night flights, especially on shorter night flights, typically <10h (Sallinen et al., 2017; Hilditch et al., 2020). This is in line with the observations of the EASA study that night flights, regardless of their duration, lead to fatigue (European Union Aviation Safety Agency (EASA), 2019). Scheduling un-augmented crews on shorter night flights, thus prohibiting BR, might increase the usage of CR. Surprisingly, CR is also used before or after BR, perhaps due to disturbed BR or planning of BR during the biological daytime of the pilot (Hilditch et al., 2020). Good quality and quantity CR can be promoted by many factors, such as the reclining angle of the seat (Roach et al., 2018). To minimize the effect of sleep inertia on performance, CR is often limited to 40–45min, with a planned 20min recovery period afterwards (European Union Aviation Safety Agency (EASA), 2012; International Air Transport Association (IATA) et al., 2015; Fatigue Countermeasures Working Group, 2018). In addition to sleep inertia, CR may pose risks to flight safety if the waking pilot does not have sufficient situational awareness. Therefore, guidelines recommend a briefing, usually including the cabin crew, prior to the use of CR, including discussions of alertness and sleep–wake history of the awake pilot, as well as an alarm system (European Union Aviation Safety Agency (EASA), 2012; Fatigue Countermeasures Working Group, 2018). In conclusion, controlled rest seems to be a viable countermeasure to manage unexpected fatigue due to sleepiness in-flight.

#### Bunk Rest

The introduction of ultra-long-range operations, with flight times exceeding 16h, indicated the need for methods to maintain crew alertness and performance. Scheduled in-flight sleep was therefore regarded as the optimal solution, but safe facilitation of in-flight sleep required the augmented crews on these flights (Flight Safety Foundation, 2005; Caldwell et al., 2009; Signal et al., 2013). BR has been shown to be beneficial to crews on longer flights, leading to better reaction times and sleepiness ratings (Eriksen et al., 2006; Gander et al., 2013b, 2014b). However, the quantity and quality of sleep obtained during BR is inferior to that obtained in a bedroom or hotel room (Signal et al., 2005, 2013; Roach et al., 2010). Studies in hypobaric chambers found that reduced cabin air pressure at cruising altitudes was not the cause of reduced sleep quality (Muhm et al., 2009). Possible factors affecting the quantity and quality of BR include environmental factors, such as comfort, noise or turbulence; psychological factors, such as difficulty taking one’s mind off the flight; and physiological factors, such as circadian timing of the nap and amount of previous sleep (Roach et al., 2011; Holmes et al., 2012; Gander et al., 2013b; Hartzler, 2014). In addition, older age is associated with a lower sleep quality (Signal et al., 2013).

It may be beneficial to provide pilots with relaxation training. Moreover, awareness of in-flight rosters may provide the most optimal rest periods for crew members (Caldwell et al., 2009; Roach et al., 2010; Hartzler, 2014).

### Stimulants

Civil aviation does not endorse the use of stimulants (i.e., pharmacological alertness-enhancing compounds), whereas military aviation allows the use of stimulants such as dextroamphetamine, caffeine and modafinil (Caldwell et al., 2009). Table 1 provides an overview of these three stimulants and their characteristics. This difference in regulations is in line with the difference in flight operations. Military flight operations tend to be more intense and unpredictable than civil operations, and cancelling a mission may jeopardize the safety of others, such as ground troops who rely on air support (Caldwell et al., 2009). Additionally, the medical oversight necessary for prescribing such stimulants is often insufficient in civil aviation, as opposed to military aviation. The efficacy of stimulants as a intervention for sustaining flight performance has been proved in several studies (Ehlert and Wilson, 2021).

The use of stimulants and their place in military operations are nevertheless subject to ethical consideration (Russo, 2007). Fatigue and subsequent reductions in performance are normal and could in theory be prevented by allowing sufficient time for sleep. Stimulants only temporarily diminish the effects of fatigue but do not alleviate these effects, making them a transient fix. Stimulants should therefore be considered a last-resort countermeasure when preventive strategies and other countermeasures have failed to mitigate fatigue (Russo, 2007; Caldwell et al., 2009).

However, a questionnaire completed by USAF fighter aircrews showed that more than half (58.6%) used stimulants at least once during a sortie (Gore et al., 2010).

Until recently the RNLAF only allowed the prescription of caffeine pills, but it is now looking to allow modafinil (Military Aviation Authority, 2021).

#### Caffeine

Caffeine is a non-prescription substance that stimulates the central nervous system by blocking adenosine receptors (Daubner et al., 2021). Its absorption through the small intestine is quick (15–40min) and its effects are noticeable within 15–20min (Caldwell et al., 2009). Its half-life is 4–6h, and it has beneficial effects in vigilance tasks as long as 8h after administration (Klopping et al., 2005). Coffee is one of the most widely used stimulants worldwide. A survey of naval aviation candidates found that 86% drank coffee daily, consistent with the percentage of the general population (Sather et al., 2017). The side effect profile is dose- and user dependent, including agitation, irritability, tremor, dysrhythmia and gastrointestinal complaints (Daubner et al., 2021). Caffeine in low dosages (<200mg, equal to approximately two cups of coffee) is generally regarded as safe, with few or no side effects reported (McMahon and Newman, 2011). In a simulator study, 200mg caffeine, administered three times with 4h intervals, elicited high levels of nauseau, jitteriness and nervousness, when compared to dextroamphetamine or modafinil (Leduc et al., 2009). Additionally, individuals taking higher daily quantities may experience withdrawal symptoms, such as headaches and muscle tremors, when caffeine intake is halted. Also, daily usage of 200–300mg caffeine can lead to tolerance, with these individual requiring a higher dosage to receive the same benefits provided to non-users by 200–300mg caffeine. Therefore, recommendations advise minimizing the daily use of caffeine, thus saving its arousal effect until really needed (Caldwell et al., 2009; Relative) contraindications for caffeine use are hypertension, hyperthyroidism, epilepsy, mania, schizophrenia, gastric, and duodenal ulcers (Daubner et al., 2021).

Although caffeine does not improve subjectively assessed sleepiness, it increases the vigilance of and performance by sleep-deprived individuals, sometimes beyond baseline levels (Kamimori et al., 2005; Doan et al., 2006; Lohi et al., 2007; Caldwell et al., 2009; McMahon and Newman, 2011). Caffeine can also counteract the impairments caused by temazepam, although this improvement seems to be dose-related up to 600mg, suggesting that multiple doses of caffeine may be necessary to maintain baseline performance levels (Wesensten et al., 2002; Kamimori et al., 2005; Klopping et al., 2005; Doan et al., 2006). Even when administered at these higher or multiple doses, the side effects of caffeine were found to be mild at most (Wesensten et al., 2005; Doan et al., 2006). Several methods of administration are available, with a cup of coffee or energy drink being the most common, but caffeine is also available in pills and chewing gum. The advantages of chewing gum are the quick absorption rate, with effects observed after 3–5min; the ease of administration; and the metered dosage (McMahon and Newman, 2011). Because caffeine is a non-prescription stimulant, it has been promoted as the optimal method of temporarily sustaining the alertness of personnel in situations with a limited level of medical oversight (Caldwell and Caldwell, 2005). The RNLAF allows the one-time use of 300mg caffeine pills as an in-flight fatigue countermeasure (Military Aviation Authority, 2021).

#### Dextroamphetamine

Dextroamphetamine is a central nervous system stimulant used during World War II to counteract fatigue by both allied and axis pilots (Rasmussen, 2011). It has a widespread dopaminergic action, binding with high affinity to dopaminergic receptors and blocking dopaminergic reuptake (Daubner et al., 2021) Dextroamphetamine reaches its peak plasma concentration in 1.5h and has an average half-life of 10h (Caldwell and Caldwell, 2005). Its most prominent effects are seen after 20 to 29h of continuous wakefulness, but may continue for up to 58h of wakefulness (Caldwell et al., 2000b, 2003a). The usual dose of dextroamphetamine is 10mg, which can be repeated every 4h (Caldwell et al., 1995, 2000b).

Due to its long history of use by the United States armed forces, the effects of dextroamphetamine have been studied in detail. However, no literature could be found of dextroamphetamine use by other countries’ militaries. Dextroamphetamine is regarded in the united States. as the drug of choice for sustaining operator alertness during prolonged periods of wakefulness (i.e., 30–70h; Caldwell and Caldwell, 2005). Dextroamphetamine was used by 65% of the pilots deployed during Operation Desert Storm, with 58–61% considering this drug to be beneficial or essential to operations (Emonson and Vanderbeek, 1995). Dextroamphetamine was also used by 97% of pilots during shorter sorties (<17h) during Operation Iraqi Freedom, with 97% of these pilots observing benefits of this drug (Kenagy et al., 2004). Dextroamphetamine has been shown to increase pilot flight performance, both in simulator and in-flight situations, a finding confirmed by a decrease in slow-waves on the electroencephalography (Collins, 1977; Caldwell et al., 1995, 1997, 2000b, 2003a; Caldwell and Caldwell, 1997). The use of dextroamphetamine was also shown to enhance pilots’ mood and ratings of vigour, and to decrease subjective feelings of fatigue and confusion (Caldwell and Caldwell, 1997; Caldwell et al., 2003a).

The side effect profile of dextroamphetamine seems to be dose-dependent. Common side effects are decreased appetite, agitation, tachycardia, hypertension, abdominal complaints, headaches and hyperactivity. Side effects which may be problematic in aviation include euphoria, tunnel vision, increased self-confidence and motor restlessness and concerns have been raised about its negative effects on pilot judgement (Hurst, 1962; Mills et al., 2001; Daubner et al., 2021). To our knowledge, however, serious problems after dextroamphetamine use have not been proven in operational personnel (Caldwell and Caldwell, 2005; Ehlert and Wilson, 2021). Its adverse effects in aviation studies seem to be limited, although jitteriness, asymptomatically elevated heart rate and increased blood pressure have been reported (Caldwell, 1996; Caldwell and Caldwell, 1997; Kenagy et al., 2004). Recovery sleep after dextroamphetamine use may be lighter and/or less restful (Caldwell et al., 2000b, 2003a; Relative) contraindications for dextroamphetamine use include symptomatic cardiovascular disease, moderate to severe hypertension, hyperthyroidism, glaucoma and psychotic disorders. Because of the possible impairing side effects, its narrow therapeutic window and its significant potential for addiction and abuse, dextroamphetamine should be used only under proper medical supervision. However, as its prescription to pilots will be functional and incidental and dextroamphetamine is not intended for daily use, the risks of addiction and abuse are thought to be low.

#### Modafinil

Modafinil {2-[(diphenylmethyl)sulfinyl]acetamide} is a relatively new wakefulness promoting stimulant that was approved for the treatment of narcolepsy in 1998. Although it’s exact mechanism of action remains undetermined, it is thought to exert a stimulating effect by altering the levels of several neurotransmitters, including serotonin, noradrenalin, dopamine and gamma-aminobutyric acid (Kim, 2012; Battleday and Brem, 2015; Daubner et al., 2021). Although modafinil is regarded primarily as a stimulant, it has significant neuroprotective effects that involve antioxidative processes (Gerrard and Malcolm, 2007) After administration modafinil is readily absorbed, reaching its maximum plasma concentrations after 2–4h with a half-life of approximately 12–15h (Robertson and Hellriegel, 2003).

Modafinil was shown to have beneficial effects on flight performance in sleep-deprived pilots (Caldwell et al., 2000a, 2004b; Leduc et al., 2009). Modafinil has been found to improve psychomotor vigilance speed, cognitive function, both subjective and objective levels of alertness as well as situational alertness, judgement and risk perception (Caldwell et al., 2004b; Wesensten et al., 2005; Killgore et al., 2008; Leduc et al., 2009; Estrada et al., 2012). Most of these studies were performed on individuals with >40h sleep deprivation. Modafinil, however, may have limited cognition enhancing properties under normal sleep conditions, and may be effective in counter-acting the effects of temazepam (Klopping et al., 2005; Kelley et al., 2011; Battleday and Brem, 2015; Daubner et al., 2021).

Headaches are the most-common described side effect of modafinil. Other common side effects include decreased appetite, tachycardia, palpitation, tremor, restlessness, dizziness, drowsiness, visual impairment, dry mouth, gastrointestinal disorders. Less common side effects include hypertension, agitation, arrythmias, Stevens-Johnson syndrome, toxic epidermal necrolysis, and Drug Rash with Eosinophilia and Systemic Symptoms (Daubner et al., 2021). The adverse effects of modafinil seen in aviation studies seem to be very limited (Wesensten et al., 2002, 2005; Klopping et al., 2005; Killgore et al., 2008; Leduc et al., 2009). Ground testing of 100mg modafinil in pilots of the Republic of Singapore Air Force found that 97.5% had no side effects, with the reported side effects being headache, anxiety, diarrhoea and insomnia (Ooi et al., 2019). However, the side effects of modafinil may be dose-related, as vertigo, nausea and dizziness were reported by most subjects after three doses of 200mg modafinil (Caldwell et al., 2000a). Although most studies have reported that recovery sleep after modafinil administration was unaffected, one study showed significant differences between modafinil and placebo in sleep quality and quantity 16h later (Walsh et al., 2004; Wesensten et al., 2005; Killgore et al., 2008; Estrada et al., 2012). In contrast to dextroamphetamine, modafinil was found to have a very low abuse potential (Bisagno et al., 2016; Relative) contraindications for modafinil use are hypertension and arrythmias.

The optimal dosage of modafinil seems to be between 100 and 200mg, which can be repeated every 4–5h (Caldwell et al., 2004b; Estrada et al., 2012). The effects of modafinil 200 and 400mg were comparable to those of 600mg caffeine, with no significant differences between the two modafinil doses (Wesensten et al., 2002). Administration of 100mg modafinil after 17, 22, and 27h without sleep to pilots subjected to 37h of continuous wakefulness resulted in these pilots maintaining flight accuracy within 15–30% of baseline levels, suggesting that three doses of 200mg might have restored flight accuracy to baseline levels (Caldwell et al., 2004b).

Modafinil has been approved as an agent to counter fatigue by the air forces of Singapore, the United States, India and France (Ooi et al., 2019). A recent observational, retrospective analysis of flight records from tactical aircraft landings on a US Navy aircraft carrier showed that pilots took modafinil during 386 (33%) of the 1,154 sorties. This drug was taken more often by more junior pilots and pilots of single seat aircrafts, with usage tending to increase later in deployment (Schallhorn, 2020).

### Fatigue Detection Technologies

Fatigue detection technologies are tools that can be effectively incorporated into overall safety management approaches. Several different technologies have been studied in order to assess their useability, of which electrocardiography and eye metrics are two major areas of interest (Hu and Lodewijks, 2020). Promising alternatives are speech analysis, which detects subtle changes in voice patterns, and photoplethysmogram sensors in the aviation headset, which detect changes in heart rate (de Vasconcelos et al., 2019; Wilson et al., 2020).

An exhaustive discussion of methods currently in development is beyond the scope of this review. The authors recommend the following papers for more information about the different modalities being investigated (Hu and Lodewijks, 2020; Bafna and Hansen, 2021; Stancin et al., 2021).

## Future Research

Despite the vast amount of literature regarding fatigue in aviation, many important topics still warrant future research. The ICAO definition of fatigue identifies work load as a possible cause of fatigue, however, the importance and effects of work load on fatigue needs to be studied more extensively. As described in the last section, the feasibility and implementation of fatigue detection technologies is another research topic warranting further research. The actual/objective incidence of fatigue in aviation, especially specified for different populations and types of operations, remains a knowledge gap. EASA has started by studying the impact of night duties on fatigue, but more research is needed to study the efficacy of regulations and how to successfully implement these. The efficacy and safety of hypnotics in aviation, especially including the newer types of hypnotics available, need more exploration. Additionally, the introduction of detailed guidelines indicating which hypnotics might be best for which situation, is necessary. Evaluations of the efficacy of pharmaceutical stimulants have found that dextroamphetamine, caffeine and modafinil have shown promise as fatigue countermeasures, but several questions remain unanswered. Foremost, the vast majority of studies have been performed in simulator environments. Replication of these studies with in-flight environments are needed to validate whether the demonstrated benefits of stimulants can be reproduced in the more chaotic and demanding situations experienced in-flight. Concerning the dosage of modafinil, most studies to date used a single dose of modafinil after longer periods of sleep deprivation, sometimes lasting >40h. By contrast, in most studies evaluating the effect of modafinil after shorter periods of sleep deprivation, several doses were administrated. The effect of a single dose of modafinil after a similar limited period of wakefulness (e.g., 24h) is particularly interesting for military aviation, as this scenario is most likely during operational missions. In most studies, participants were asked to completely discontinue their daily caffeine intake for the duration of the experiments. While this increases the methodological strength of controlled studies, it does not reflect real-world practice, in which individuals, especially military aviators on deployment, regularly consume caffeine. The synergetic effects of modafinil and dextroamphetamine combined with caffeine, and the relevance of this combination for (military) aviation, are therefore unknown. Moreover, any negative effects of modafinil and dextroamphetamine on sleep quality and quantity, with or without caffeine, should be unraveled, as military operations require pilots to be fit to fly the next mission as soon as reasonably possible. Lastly, these findings should be tailored into airframe- and mission-specific profiles, as helicopter operations differ substantially from high performance aircraft operations.

## Discussion

Fatigue remains an important safety risk in both civil and military aviation. Possible causes of fatigue include sleep loss, extended time awake, circadian rhythm disruption and work load. Despite regulations limiting flight times and suggesting optimal rosters, not all effects of fatigue are mitigated, especially in military operations, where limits may be extended due to operational necessities. Thus, it is impossible to rely solely on regulations to prevent fatigue.

The one and only true prevention of fatigue is a good night’s sleep. However, the demanding aviation environment often leads to sleep loss (both quantity and quality), especially when sleeping away from home, such as during layovers and on military deployments. Consequently, restoring sleep through pre- or in-flight naps is considered to be the most beneficial countermeasure, as this not only (partly) alleviates sleep loss but also reduces sleep pressure by shortening the period of extended wakefulness. The use of hypnotics is prohibited by many civil aviation organizations, although it is allowed by many military organizations. Hypnotics are therefore regularly prescribed to facilitate sleep, especially during military deployments when transient factors may impede good quality and quantity sleep. Stimulants, authorized in military aviation, do not alleviate the fatigue itself, but only temporarily diminish its effects. Stimulants should therefore be considered a last-resort countermeasure and should only be used when all other countermeasures do not suffice. Dextroamphetamine has great potential in increasing flight performance after sleep loss, but its significant abuse potential is a reason for concern. Caffeine is widely available and globally appreciated for its stimulating effects and does not require extensive levels of medical oversight. However, a higher daily intake of caffeine may induce tolerance, with customary dosages having reduced effects. Modafinil is a relatively new stimulant, with an efficacy comparable to that of caffeine in restoring flight performance, although it is better at improving subjective feelings of sleepiness and has a longer duration of effects than caffeine. Modafinil may therefore be a good choice of stimulant when caffeine does not work sufficiently or when a longer duration of increased alertness is needed. However, many questions regarding modafinil remain to be answered, such as the effect of a single dose on a limited period of wakefulness, the interaction between daily caffeine intake and modafinil administration, and the potential negative effects of modafinil on subsequent sleep. Future research should assess the effects of modafinil in operationally realistic scenarios, account for its possible synergetic effects with caffeine and evaluate its possible subsequent effects on sleep.

## Author Contributions

YW-J conceptualized this paper and performed the search and critically appraisal of the literature and was involved in drafting and revision of the manuscript. TW performed the search and critically appraisal of the literature and was involved in drafting and revision of the manuscript. WR was involved in drafting and revision of the manuscript. JR conceptualized this paper and was involved in drafting and revision of the manuscript. All authors contributed to the article and approved the submitted version.

## Conflict of Interest

The authors declare that the research was conducted in the absence of any commercial or financial relationships that could be construed as a potential conflict of interest.

## Publisher’s Note

All claims expressed in this article are solely those of the authors and do not necessarily represent those of their affiliated organizations, or those of the publisher, the editors and the reviewers. Any product that may be evaluated in this article, or claim that may be made by its manufacturer, is not guaranteed or endorsed by the publisher.
